# Evaluation of *Phaseolus vulgaris* *lec‐* *lpa* as Alternative Protein Source on Growth Performances, Health Status, Faecal Minerals and Gut Microbiota in Weaned Piglet's Diet

**DOI:** 10.1002/vms3.70597

**Published:** 2025-09-15

**Authors:** Benedetta Canala, Sara Frazzini, Matteo Dell'Anno, Matteo Santoru, Irene Ferri, Francesca Sparvoli, Lucrezia Luciani, Bianca Castiglioni, Paola Cremonesi, Filippo Biscarini, Martina Ghidoli, Roberto Pilu, Luciana Rossi

**Affiliations:** ^1^ Department of Veterinary Medicine and Animal Sciences – DIVAS Università degli Studi di Milano Lodi Italy; ^2^ Institute of Agricultural Biology and Biotechnology Consiglio Nazionale delle Ricerche Milan Italy; ^3^ Institute of Agricultural Biology and Biotechnology Consiglio Nazionale delle Ricerche Lodi Italy; ^4^ Department of Agricultural and Environmental Sciences‐Production Landscape, Agroenergy Università degli Studi di Milano Milan Italy

**Keywords:** alternative protein source, common bean, faecal minerals, microbiota, organic farming, soybean

## Abstract

**Background:**

Genetically modified soybean is largely used in animal feed and its massive cultivation affects the environmental sustainability of livestock and the dependency for the import in the European market.

**Objectives:**

The aim of this study was to evaluate the partial substitution of soybean meal with an innovative common bean genotype (*Phaseolus vulgaris lec‐lpa*) with reduced content of anti‐nutritional factors on zootechnical performance, gut microbiota modulation and faecal minerals in post‐weaning piglets.

**Methods:**

Fourteen piglets were divided into a control group fed with a basal diet and a treatment group fed with a commercial diet in which 7.3% of soybean meal and 0.8% of soybean oil were replaced with 10% of *P. vulgaris lec‐ lpa* for 28 days. BW, ADG, ADFI and FCR were evaluated, and diarrhoea incidence was recorded. Evaluation of pH, nitrogen content, protein digestibility and mineral content was performed on faecal samples. Microbiota was analysed by rectal swabs samples. Blood serum metabolic profile was evaluated.

**Results:**

The treatment group showed lower BW and ADG during the trial (*p* < 0.05), but the health status of the animals was preserved. The treatment group released lower levels of minerals in faeces when compared with the control group after 28 days (*p* < 0.05) suggesting a lower dispersion of faecal minerals in the environment. Significant Beta diversity index was observed at 14 and 28 days (*p* < 0.05). *Roseburia* and *Butyricicoccus* increased in treatment group at day 28 (*p* < 0.05). These genera are associated with SCFA production, contributing to the maintenance of intestinal integrity, promoting positive bacterial populations and limiting inflammatory phenomena.

**Conclusions:**

In conclusion, *P. vulgaris lec‐ lpa* could be a viable and sustainable alternative protein source to reduce the European protein gap, playing a potential role in microbiota modulation and faecal minerals release.

## Graphical abstract

Partial replacement of soybean meal with an innovative *Phaseolus vulgaris* genotype with low lectin and phytic acid content was evaluated in weaned pigs’ diet. Although piglets fed the bean‐based diet showed reduced growth performance, their health status was preserved, registering lower faecal mineral excretion, suggesting reduced environmental dispersion. Gut microbiota analysis revealed increased abundance of *Roseburia* and *Butyricimonas*, genera linked to short‐chain fatty acid production, highlighting potential benefits for intestinal integrity and microbial balance.

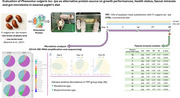

## 1 Introduction

According to the United Nations, the world population will reach 10 billion in 2050 (United Nations 2022). For this reason, there will be an increase in demand for animal products, and it will be necessary to develop a more sustainable livestock system. Animal feed is typically formulated mainly using cereals and other ingredients, including soybean meal and agro‐industrial by‐products, to ensure an optimal nutrient balance at an affordable cost for the farmer. Soybean is the main plant‐based protein source for animal feed, with no exception for pig farms where a balanced diet and adequate protein intake are required (Panagiota et al. 2014). However, the conspicuous use of soybean meal in animal feed has led to negative impacts: the European market dependency on soybean imports (Zmudzińska et al., 2020), a loss of biodiversity, soil depletion and increased pollution due to a massive cultivation (Fang and Kong, 2022). Among the alternative sources, legumes are possible candidates due to their higher content in protein (Wang et al. 2022), in particular the common bean (*Phaseolus vulgaris*). However, their use in pig diets is generally limited due to the presence of several well‐documented anti‐nutritional factors (ANFs), such as lectins, trypsin inhibitors, tannins and phytic acid (Huisman et al. 1990). These compounds can impair nutrient digestibility, impair gut function and reduce growth performance, especially in piglets. In most feed formulations, the inclusion of common beans is restricted to less than 5%, depending on availability and price, because of these limitations. Nevertheless, the anti‐nutritional factors of legumes inclusion in animal diets are well established, resulting in the discovery of protease inhibitors, saponins, lectins, and alkaloids, which may impair digestion or feed intake in pigs (Jezierny et al. 2010; Woyengo et al. 2017; Wang et al. 2022). In more detail, lectins, such as common bean phytohemagglutinin, are molecules able to bind carbohydrates of glycoproteins present on the cell surface of the gut epithelium (Wiesinger et al. 2022), causing atrophy of microvilli resulting in a reduction of normal intestinal function (Ramadass et al. 2010; Yamamoto et al. 2010; Kong et al., 2022) and pancreatic hyperplasia (Rocha et al. 2014; Vojdani et al. 2020). Another important anti‐nutritional factor in legumes is phytic acid, also known as myo‐inositol‐1, 2, 3, 4, 5, 6‐hexakisphosphate (InsP6), which acts as a strong chelator of mineral cations (Sparvoli and Cominelli, 2015). The lack of phytase in monogastric animals leads to a poor digestion of phytic acid, resulting in the excretion of phytate salts, leading to limited phosphorus and mineral availability for the organism. Moreover, phytic acid is scarcely hydrolysed by pigs and can bind to dietary nutrients, reducing their digestibility. This led to a decrease in the digestibility with a higher release of unabsorbed nutrients with faeces.

Several studies have evaluated the partial substitution of soybean with common bean. In detail, in the study of Myer et al. (1982), the inclusions of 5% and 15% of common bean in young pig diets were tested, and the results confirmed the impact of anti‐nutritional factors, such as reduction in zootechnical performances, decrease in digestibility and organ weight alteration. Furthermore, different papers have demonstrated that phytic acid can increase endogenous nutrient losses in pigs and poultry (Woyengo and Nyachoti 2013). Moreover, undigested ingredients can contribute to the development of gastrointestinal disorders that often affect the weaning period (Dell'anno et al. 2020). Thus, the role of nutrition and microbiota eubiosis is particularly relevant for pathology prevention and antibiotic reduction in swine (Saettone et al. 2020). To overcome these limitations, efforts have been made to reduce the ANF content in legumes through plant breeding. However, *Phaseolus vulgaris* is known to be recalcitrant to genetic transformation and biotechnological modification, making the development of improved lines particularly challenging (Moura et al. 2024). Despite this, progress has been achieved through classical mutagenesis and selection. In recent years, a mutant line of common bean has been identified and characterised for its significantly reduced levels of both phytic acid and lectins, while retaining agronomic viability (Campion et al. 2009; Campion et al. 2013; Sparvoli et al. 2016; Sparvoli et al. 2021). This mutant line, carrying a low phytic acid (*lpa*) and low‐lectin phenotype, represents a potentially valuable alternative protein source in pig nutrition. The *lpa* trait may enhance phosphorus bioavailability and reduce the environmental impact of manure through lower phosphorus excretion (Raboy, 2020), while reduced lectin content may alleviate gastrointestinal side effects typically associated with common bean inclusion. However, the mutant is not entirely free from anti‐nutritional factors, and its effectiveness and safety must be evaluated in vivo to understand its role in animal nutrition. Considering that, the bioavailability of nutrients is a key point not only for animal nutrition but also for public and environmental issues (Byrne and Murphy 2022). In fact, the excreted phytate salts negatively impact soil and contribute to water pollution (Campion et al. 2009; Hejna et al. 2020). Nowadays, according to the agroecology principles, the European Union has implemented several measures to control the presence of heavy metals in the environment resulting from human activities, including livestock (Hejna et al. 2018).

Considering this background, the present study investigated the effects of a recently developed mutant line of common bean (*Phaseolus vulgaris* L.), with reduced levels of phytic acid and lectins (Campion et al. 2009; Campion et al. 2013; Sparvoli et al. 2016; Sparvoli et al., 2021), when included in a complete‐balanced diet for post‐weaning piglets. The aim of this study was to assess the effect of *Phaseolus vulgaris* mutant meal dietary inclusion on mineral bioavailability and gut microbiota modulation in post‐weaned piglets.

## 2 Materials and Methods

### 2.1 Animals Housing and Experimental Design

The experimental trial was conducted after the ethical authorisation of the Animal Welfare Organisation of the University of Milan (OPBA authorisation no. 4_2023) and in accordance with European regulations (European Parliament and Council 2010) at the Experimental Zootechnical Centre of the University of Milan (Lodi, Italy). 14 weaned piglets (Landrace x Large White; 28 days ± 2), individually identified by ear tags, were housed in six different pens (3 pens/group). Considering the ethical authorisation aimed at studying the effect of common bean dietary inclusion under in‐field conditions, piglets were allotted in groups in accordance with animal welfare regulations (European Parliament and Council 2010; D Lgs, 2014). In each group 7 piglets were housed as follows: 4 animals in 2 pens (2 piglets/pen) and 3 animals in 1 pen (3 piglets/pen). Piglets were divided into two experimental groups homogeneous for sex (50% male– 50% female) and weight (7.4 ± 0.01 kg) and kept under standard environmental conditions (28°C and 60%–70% relative humidity) for 28 days. Environmental enrichment was added to the pens to ensure animal welfare and ethogram manifestation (D. Lgs, 2014). Groups were differentiable by the provided diet: the control group (CTRL: 7 piglets, 3 pens) fed a commercial diet and the treatment group (TRT) (TRT: 7 piglets, 3 pens) fed a commercial diet in which 7.3% of soybean meal, 0.8% of soybean oil and 1.9% of barley were replaced with 10% low lectin and low phytic acid common bean meal (*P. vulgaris* *lec‐* *lpa*). The two diets, provided ad libitum, were formulated to be isoenergetic and isonitrogenous using Plurimix software (Fabermatica, Cremona, Italy) in line with the nutritional requirements of post‐weaning piglets (National Research Council 2019) and supplied by Ferraroni Spa (Bonemerse, Italy) (Table 1). Animals had unlimited access to water through nipple drinkers (2 nipples/pen) and feed, in addition the piglets’ health status was monitored twice per day.

**TABLE 1:** Experimental diets composition and principal chemical characteristics of control (CTRL, fed basal diet) and treatment group (TRT, fed basal diet with 10% of *P. vulgaris* *lec‐* *lpa*). All data are expressed as percentage as fed basis.

	CTRL	TRT
**Ingredients, % (as fed basis)**		
Barley, meal	23.99	22.09
Wheat, meal	17.01	17.01
Wheat, flakes	9.99	9.99
Fermented soy concentrate	7.02	7.02
Corn, flakes	5.67	5.67
Milk whey‐fat, blend 50%	4.95	4.95
Corn, meal	4.50	4.50
Milk whey	3.96	3.96
Dextrose monohydrate	2.97	2.97
Beet pulp	1.98	1.98
Soybean protein concentrate	1.62	1.62
Plasma, meal	1.53	1.53
Soybean, oil	1.80	1.00
Acidifiers mix^a^	0.80	0.80
L‐lysine	0.62	0.62
Coconut oil	0.60	0.60
Dicalcium phosphate	0.50	0.50
Dietary fibres	0.50	0.50
Harring, meal	0.50	0.50
Vitamins and mineral elements^b^	0.50	0.50
DL‐methionine	0.28	0.28
Calcium benzoate	0.25	0.25
L‐treonine	0.25	0.25
Bentonite	0.25	0.25
Tri‐di butyrin	0.20	0.20
Sodium chloride	0.15	0.15
L‐valine (96.5%)	0.11	0.11
Calcium carbonate	0.05	0.05
+ Enzymatic Mix^c^	0.05	0.05
L. tryptophan	0.05	0.05
Flavour	0.05	0.05
Soybean, meal (42.8% CP)	**7.30**	—
*Phaseolus vulgaris* *lec–* *lpa*, meal	—	**10.00**
**Calculated chemical composition** ^d^		
Crude protein (%)	17.51	16.75
Ether extract (%)	6.60	6.68
Crude fibre (%)	3.10	2.85
Digestible energy (Mc/Kg)^e^	3580	3570
K (%)	0.60	0.60
Cl (%)	0.41	0.41
Ca (%)	0.40	0.40
P (%)	0.40	0.40
Na (%)	0.20	0.20
S (%)	0.20	0.20
Mg (%)	0.08	0.08

^a^ Citric acid 0.17 mg, Calcium formate 89.60 mg, Fumaric acid 28.00 mg.

^b^ Additives per kg: Vitamin A (Retynil Acetate) 16,000 IU, Vitamin D3 (Cholecalciferol) 2,000 IU, Betaine hydrochloride 1,120 mg, Biotin 0.22 mg, Choline chloride 336.00 mg, Folic acid 1.12 mg, Niacinamide 33.60 mg, Calcium D‐pantothenate 16.80 mg, Vitamin B1 (Thiamine mononitrate) 2.24 mg, Vitamin B12 (Cyanocobalamin) 0.03 mg, Vitamin B2 (Riboflavin) 5.60 mg, Vitamin B6 (Pyridoxine hydrochloride) 3.92 mg, Vitamin C (Sodium Calcium Ascorbyl Phosphate) 168.00 mg, Vitamin E (Alpha‐tocopheryl acetate) 134.40 mg, Vitamin K3 (Menadione nicotinamide bisulphite) 2.24 mg, Copper (copper oxide‐I) 100 mg, Iodine (Calcium iodate anhydrous) 1.12 mg, Iron (Iron‐II sulphate monohydrate) 134.40 mg, Manganese (Manganese‐II oxide) 56.00 mg, Selenium (Sodium selenite) 0.39 mg, Zinc (Zinc oxide) 112.00 mg, L‐lysine monohydrochloride, technically pure 5,382 mg, L‐threonine 2,758 mg, L‐tryptophan 480 mg, Propyl gallate 0.17 mg, Bentonite 2,746.02 mg, Sepiolite 645.68 mg, Butyl hydroxytoluene (BHT) 0.34 mg.

^c^ phytase (solid form) 2, 016 FTU, Endo1,3(4)‐beta‐glucanase 1,650 UV, Endo‐1,4‐beta‐xylanase 1,210 UV, Benzoic acid 1,380,40 mg.

^d^ Calculation was carried out with Plurimix Software  (Fabermatica, Cremona, Italy).

^e^ Digestible Energy (Mc/kg) content was estimated by NRC (2012).

### 2.2 Chemical Evaluation and Mineral Composition of Experimental Diets

According to the official methods of analysis (AOAC 2023), the chemical evaluation of the experimental diets was conducted on dry matter (DM), ether extract (EE), crude protein (CP), crude fibre (CF) and ash content. DM was evaluated by drying samples in a forced‐air oven at 65°C for 24 h (AOAC method 930.15). The determination of EE was performed using ether extraction according to AOAC method 2003.05. CP content was determined using the Kjeldahl method (AOAC method 2001.11). The AOCS method Ba 6a‐05 was performed to evaluate the CF content. Ash content was evaluated after sample incineration at 550°C for 3 hours according to AOAC method 942.05. The mineral composition of the feed was performed by inductively coupled plasma mass spectrometry (ICP‐MS). Calibration curves were used for each element (Na, Mg, Al, P, K, Ca, Cr, Mn, Ni, Cu, Zn, Cd). Samples (0.3 g) were dried and then digested in 10 mL of 65% HNO3 using Teflon tubes and then heated at a one‐step temperature ramp (at 120°C in 10 min and maintained for 10 min). Once mineralised, the samples were cooled for 20 min and then tested in polypropylene test tubes. The mineralised sample was diluted with 0.3 M HNO3 in MilliQ water at a ratio of 1:100. An ICP‐MS (BRUKER Aurora‐M90 ICP‐MS) measured the concentration of elements. The check of nebulisation performance was performed by adding an aliquot of 2 mg/L of a standard solution (72Ge, 89Y, 159 Tb) to the samples up to reach a final concentration of 20 µg/L. In order to remove the polyatomic interference, a collision‐reaction interface (CRI) with an H2 flow of 80 mL/min through a skimmer cone was used (Giupponi et al. 2021).

### 2.3 Zootechnical Performances, Health Status and Sample Collection

Piglets were individually weighed at days 0, 7, 14 and 28. Feed refuse was weighed three times a week, and average daily gain (ADG), average daily feed intake (ADFI) and feed conversion ratio (FCR) were calculated. Faecal samples were collected at days 0, 14 and 28 for microbiological and mineral content analysis. The health status of the animals was evaluated by faecal consistency using a four‐level scale: 0 = normal consistency (faeces firm and well formed), 1 = soft consistency (faeces soft and formed), 2 = mild diarrhoea (fluid faeces, usually yellowish) and 3 = severe diarrhoea (faeces watery and projectile) (Rossi et al. 2023). A faecal score ≤ 1 was considered normal, on the contrary a faecal score >1 was defined as diarrhoea. The colour of the faecal samples was recorded according to a three‐level scale: 0 = yellowish colour, 1 = greenish colour and 2 = brown colour. The score ≥ 2 was considered normal (Rossi et al. 2014). Rectal swabs were collected on days 0, 14 and 28 for microbiota analyses and stored at ‐80°C to maintain DNA integrity. Blood samples were collected from the jugular vein using vacuum tubes without anticoagulant at the beginning and at the end of the trial.

### 2.4 Blood Serum Analyses of Metabolites and Minerals

Blood serum samples were collected at day 0 and day 28 of the trial. 10 mL vacuum tubes were centrifuged at 3000 rpm for 15 min at 4°C in order to recover the serum and perform the analyses, using a multiparametric autoanalyzer for clinical chemistry (ILab 650; Instrumentation Laboratory Company, Lexington, MA, USA). The analysed serum parameters were calcium (mmol/L), alanine aminotransferase (ALT‐GPT; IU/L), total protein (g/L), albumin (g/L), globulin (g/L), albumin/globulin (A/G ratio), urea (mmol/L), glucose (mmol/L), aspartate aminotransferase (IU/L), total bilirubin (µmol/L), total cholesterol mmol/L), phosphorus (mmol/L), magnesium (mmol/L) and alkaline phosphatase (IU/L). The analysis was performed by the Experimental Zooprophylactic Institute of Lombardy and Emilia Romagna (IZSLER, Brescia, Italy).

### 2.5 pH, Minerals, Nitrogen Content and Protein Digestibility of Faecal Samples

The pH evaluation was performed using a pH 7 Vio portable pH meter (XS instruments, Carpi, Italy). After instrument calibration, the pH meter electrode was directly put on faecal samples collected on days 0, 14 and 28 of both groups. The resulting values were recorded, and the average and standard deviation were calculated.

The mineralisation of faecal samples, collected at days 0 and 28, was obtained by inductively coupled plasma mass spectrometry (ICP‐MS). Calibration curves were used for each element (Na, Mg, Al, K, Ca, Cr, Mn, Fe, Co, Ni, Cu, Zn, As, Se, Mo, Cd, Pb, P). After samples were dried, the procedure was performed as previously described. Faeces collected at day 0 and day 28 were analysed in order to determine the nitrogen content and digestibility.

For the evaluation of nitrogen content, samples were analysed with the Kjeldahl method (AOAC method 2001). The apparent protein digestibility was determined through the acid insoluble ash (AIA) marker (Alvarez‐Rodriguez et al. 2016) in feed and faecal samples. At the end of the protocol, the apparent total tract nitrogen digestibility (ATTD) of protein was calculated using the formula below:

$$\mathrm{ATTD}\;\left(\%\right)=100\times \left[1-\left(\frac{\mathrm{marker}\;\mathrm{in}\;\mathrm{feed}}{\mathrm{marker}\;\mathrm{in}\;\mathrm{faeces}}\times \frac{\mathrm{nutrient}\;\mathrm{in}\;\mathrm{faeces}}{\mathrm{nutrient}\;\mathrm{in}\;\mathrm{feed}}\right)\right]$$

### 2.6 Microbiota Analysis: Bacterial DNA Extraction, V3–V4 Region Amplification and Sequencing

The DNA extraction from rectal swabs was performed using the QIAamp Power Faecal Pro DNA Kit (Quiagen GmbH, Hilden, Germany) following the manufacturer's instructions. The extracted DNA samples were quantified using a NanoDrop ND‐1000 spectrophotometer (NanoDrop Technologies, Wilmington, DE, USA). Bacterial DNA was amplified using specific primers, according to J. G. Caporaso et al. (2011), for V3‐V4 regions of the 16S rRNA gene.PCR was performed on each sample in 25 µL as a final volume. A total volume of 12.5 µL of KAPA HIFI Master MIX 2x (Kapa Biosystems, Inc., MA, USA) and 0.2 µL of primers (100 µM) were added to 2 µL of DNA (5 ng µL^−1^). Also, control samples were prepared. Amplifications were performed in an Applied Biosystem 2700 thermal cycler (ThermoFisher Scientific). The libraries were prepared using the 16S Metagenomic Sequencing Library Preparation Protocol (Illumina, San Diego, CA, USA), quantified by Real Time PCR with KAPA Library Quantification Kits (Kapa Biosystems, Inc., MA, USA) and sequenced in one MiSeq (Illumina, San Diego, CA, USA) run with 2×250‐base paired‐end reads. Demultiplexed paired‐end reads from the 16S rRNA sequencing were initially assessed for quality with FastQC (Andrews, 2010). Primer and adapter sequences were removed with Cutadapt (Marcel, 2011), while low‐quality bases were trimmed with Sickle (Joshi & Fass, 2011), applying a Phred score cutoff of >20 (reads were trimmed at the end when quality dropped below this threshold). The cleaned forward and reverse reads were subsequently merged with the Python‐based pipeline MICCA (Microbial Community Analysis), using the “merge pairs” function with default parameters (minimum overlap length of 32 bases and up to 8 mismatches allowed in the overlap region).Merged reads were then quality‐filtered, discarding sequences containing ambiguous bases or with an expected error rate exceeding 1% (i.e., more than one error every 100 nucleotides). The remaining sequences were processed to infer Amplicon Sequence Variants (ASVs) through the denoising algorithm (denovo_unoise) implemented in the MICCA “otu” function (Rosen et al., 2012). Taxonomic assignment of the resulting Operational Taxonomic Units (OTUs) was performed with the MICCA “classify” function, using the RDP 16S rRNA database (Wang et al., 2007) and applying a minimum confidence threshold of 0.8. ASVs represented by fewer than 15 counts in fewer than three samples were excluded from the final ASV table.Alpha diversity was estimated in terms of richness (Chao1 and ACE indices), diversity (Shannon, Simpson, and Fisher’s alpha indices), and evenness (Simpson E and Pielou’s J). Beta diversity across samples was calculated using the Bray–Curtis dissimilarity metric. Prior to Bray–Curtis calculations, OTU abundances were normalized to account for unequal sequencing depth through cumulative sum scaling (CSS).

### 2.7 Statistical Analysis

Results of zootechnical performances, faecal protein content and protein digestibility were analysed with GraphPad Prism 9.0 software (San Diego, CA, USA) using a generalised linear model (two‐ways ANOVA) considering the effect of treatment (Trt), time (Time) and the interaction treatment‐time (Trt x Time). For the evaluation of ADFI and FCR, the pen was considered as the experimental unit. For the other parameters, an individual piglet was considered as the experimental unit. Faecal scores were converted to a dichotomous variable (normal/pathological), considering diarrhoea for a faecal score > 1, moderate diarrhoea for a score = 2 and severe diarrhoea for a score = 3. Covariance matrix analysis was performed for serum metabolites and mineral composition of faeces using JMP Pro 16 (SAS Inst. Inc., Cary, NC, USA). The multivariate analysis of principal component analysis (PCA) was used to plot the data of faecal mineral content of 28 days of trial. For the gut microbiota analysis, differences in alpha diversity indices between groups were evaluated using a linear model including the fixed effects of time and treatment. Pairwise comparisons between groups, as well as differences in community composition based on Bray–Curtis dissimilarities, were tested by permutational multivariate analysis of variance (PERMANOVA) using the adonis function of the vegan package in R (Oksanen et al., 2020). Statistical significance was set at p ≤ 0.05, and results are reported as means ± standard error or standard deviation.

## 3 Results

### 3.1 Chemical Evaluation of Experimental Diets

The nutritional profile revealed comparable values in terms of moisture, CP, EE, CF and ash. Data in Table 2 showed that the inclusion of 10% of *P. vulgaris* *lec‐* *lpa* did not affect the main nutrient composition of the treatment diet.

**TABLE 2:** Analysed chemical and mineral compositions of experimental diets of control (CTRL) and *P. vulgaris* *lec‐* *lpa* (TRT) groups.

	CTRL	TRT
**Analyte**		
Moisture	6.06 ± 0.19	6.84 ± 0.09
CP	16.44 ± 1.59	16.37 ± 0.29
EE	6.51 ± 0.13	6.76 ± 0.13
CF	3.00 ± 0.26	3.13 ± 0.52
Ash	5.29 ± 0.41	4.83 ± 0.01
**Mineral composition**		
Na g/kg	1.81 ± 0.10	1.95 ± 0.12
Mg g/kg	1.75 ± 0.18	1. 76 ± 0.24
Al g/kg	0.29 ± 0.03	0.30 ± 0.01
P g/kg	4.90 ± 0.65	4.79 ± 0.39
K g/kg	8.34 ± 0.75	7.59 ± 0.37
Ca g/kg	0.62 ± 0.02	0.60 ± 0.07
Cr mg/kg	1.08 ± 0.29	1.08 ± 0.08
Mn mg/kg	88.63 ± 12.66	79.45 ± 11.54
Ni mg/kg	1.76 ± 0.41	1.79 ± 0.81
Cu mg/kg	103.30 ± 3.08	131.20 ± 23.37
Zn mg/kg	49.81 ± 13.57	58.04 ± 24.47
Cd mg/kg	0.01 ± 0.01	0.09 ± 0.02

*Note*: Data are presented as means ± standard deviation.

Abbreviations: CF, crude fibre; CP, crude protein; EE, ether extract.

### 3.2 Zootechnical Performances and Diarrhoea Incidence

**FIGURE 1:**
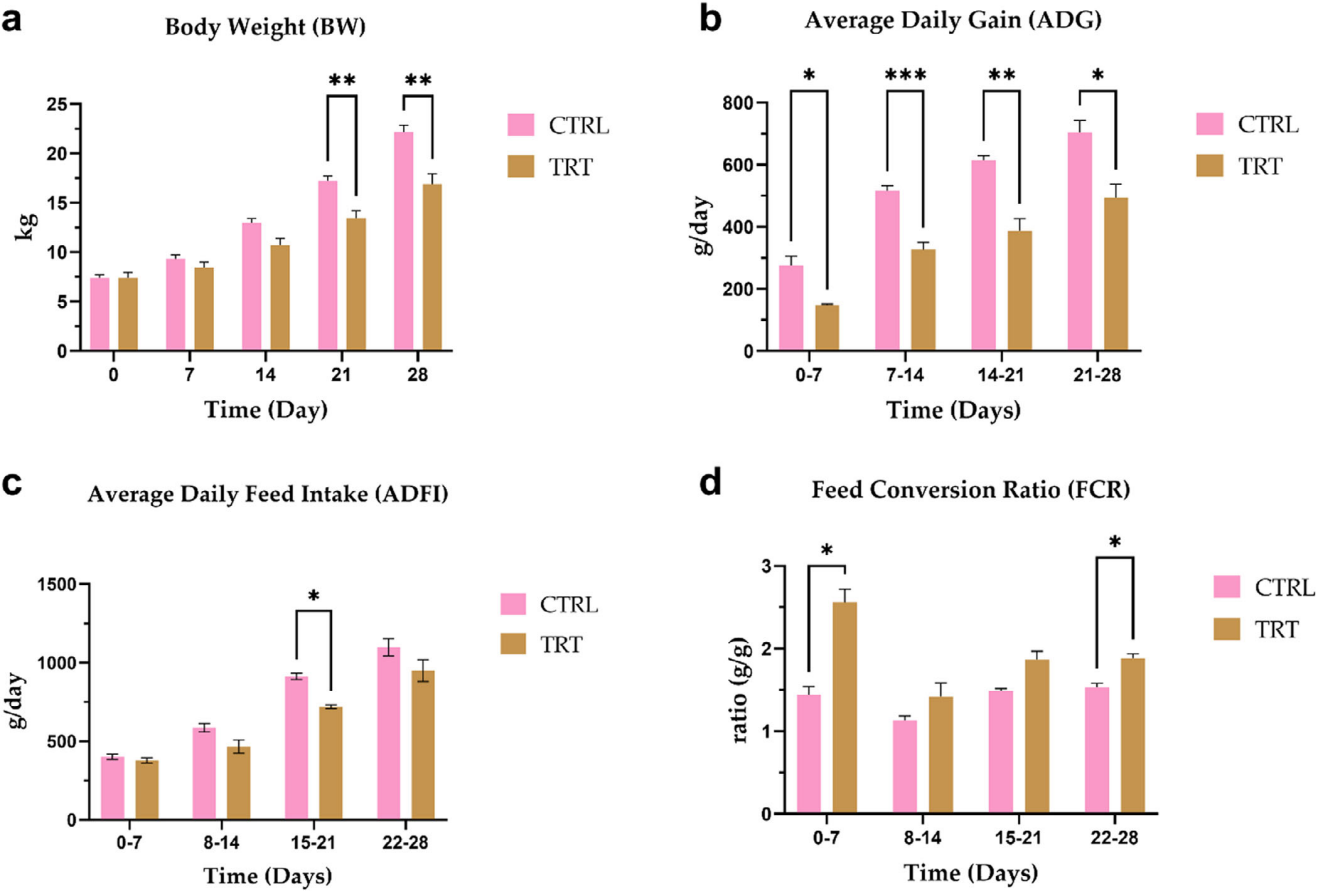
Zootechnical performances of control (CTRL) and *P. vulgaris* *lec‐* *lpa* (TRT) groups during the 28 days of experimental trial. (a) Body weight at day 0, 7, 14, 21 and 28; (b) average daily gain at day 0–7, 7–14, 14–21 and 21–28; (c) average daily feed intake at day 0–7, 8–14, 15–21 and 22–28; (d) feed conversion ratio at day 0–7, 8–14, 15–21 and 22–28. Asterisks indicates statistically significant differences between groups (* *p* < 0.05, **: *p* < 0.01, ***: *p* < 0.001). Data are presented as means ± standard error.

Results of zootechnical performance showed significant differences during the 28 days of trial (*p* < 0.05) (Figure 1). Body Weight (BW) revealed a significant increase in the CTRL group at day 21 (*p* < 0.01) and day 28 (*p* < 0.01) compared to the TRT group. Consequently, the ADG was significantly higher from 7 to 28 days of trial in the CTRL compared to the TRT group (*p* < 0.05). ADFI showed a significant increase during the week from 15 to 21 days of trial in CTRL compared to TRT (*p* < 0.05). FCR registered a significant decreased ratio in CTRL from 0 to 7 and from 22 to 28 days compared to TRT (*p* < 0.05).

At the arrival (day 0), two animals in the CTRL group and one piglet in TRT showed diarrhoea occurrence. From day 7, up to the end of the trial, no diarrhoea cases were reported in both experimental groups. At day 0, only one animal in the control group with clinical signs of diarrhoea showed a greyish colour of the faeces. From day 7 up to the end of the trial, all piglets exhibited normal faecal colour.

### 3.3 pH, Mineral Content in Faeces and Apparent Total Tract Digestibility of Protein

Data in Table 3 did not show significant differences in faecal pH values among groups during the trial.

**TABLE 3:** Faecal pH measured at day 0, 14 and 28 in control (CTRL) and *P. vulgaris* *lec‐* *lpa* (TRT) groups.

Time (day)	CTRL	TRT	SE
0	6.66	6.57	0.1203
14	6.08	6.27	0.1203
28	6.16	6.19	0.1203

*Note*: Data are presented as means and standard error (SE).

**FIGURE 2:**
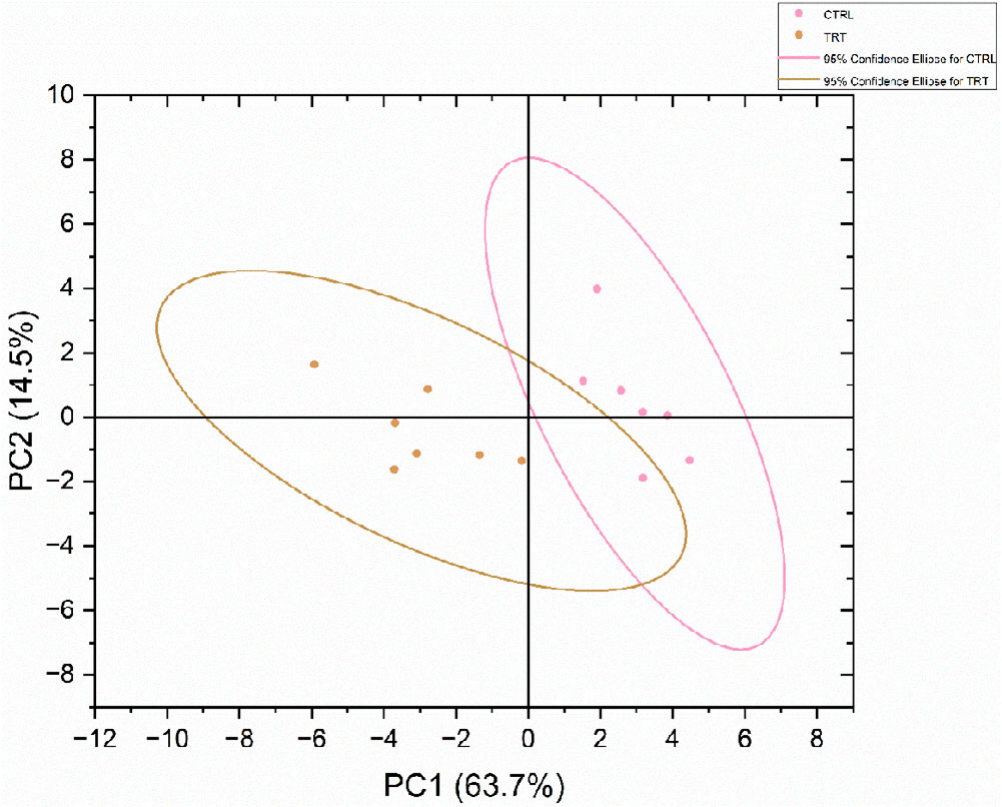
Principal component analysis (PCA) of faecal minerals in control (CTRL) and *P. vulgaris* *lec‐* *lpa* (TRT) groups at day 28. PC1: principal component 1 and PC2: principal component 2.

The following minerals: Mg, Al, P, Ca, Cr, Mn, Fe, Co, Ni, Cu, Zn, Se, Cd and Pb were statistically lower in *P. vulgaris lec‐lpa* group (TRT) compared to the CTRL (*p* < 0.02) (Table 4). PCA results showed a separate clustering of CTRL and TRT groups after 28 days for their faecal mineral content (Figure 2).

**TABLE 4:** Mineral content in control group (CTRL) and *P. vulgaris* *lec‐* *lpa* (TRT) faeces. Statistically significative values with *p* < 0.05.

Mineral (as dry matter basis)	Unit	CTRL	TRT	SE	p‐value
Na	g/kg	0.92	0.57	0.13	0.0854
Mg	g/kg	10.04^a^	7.33^b^	0.24	< 0.0001
Al	g/kg	1.63^a^	1.44^b^	0.04	0.0052
P	g/kg	8.49^a^	6.69^b^	0.41	0.0118
K	g/kg	12.35	10.94	0.76	0.2396
Ca	g/kg	6.51^a^	4.85^b^	0.41	0.0155
Cr	g/kg	6.93^a^	6.18^b^	0.18	0.0158
Mn	g/kg	0.68^a^	0.49^b^	0.02	< 0.0001
Fe	g/kg	2.10^a^	1.74^b^	0.07	0.0094
Co	g/kg	1.03^a^	0.73^b^	0.03	< 0.0001
Ni	g/kg	7.55^a^	5.43^b^	0.26	0.0003
Cu	g/kg	0.93^a^	0.77^b^	0.03	0.006
Zn	g/kg	0.81^a^	0.63^b^	0.02	< 0.0001
As	g/kg	0.41	0.36	0.02	0.0655
Se	g/kg	1.46^a^	1.07^b^	0.10	0.0184
Mo	g/kg	1.40	1.28	0.17	0.6161
Cd	g/kg	0.32^a^	0.26^b^	0.01	0.0016
Pb	g/kg	0.91^a^	0.77^b^	0.03	0.0119

*Note*: Data are presented as means and standard error (SE).

^a,b^Means with diverse lowercase letters are significantly different (*p* < 0.05).

**FIGURE 3:**
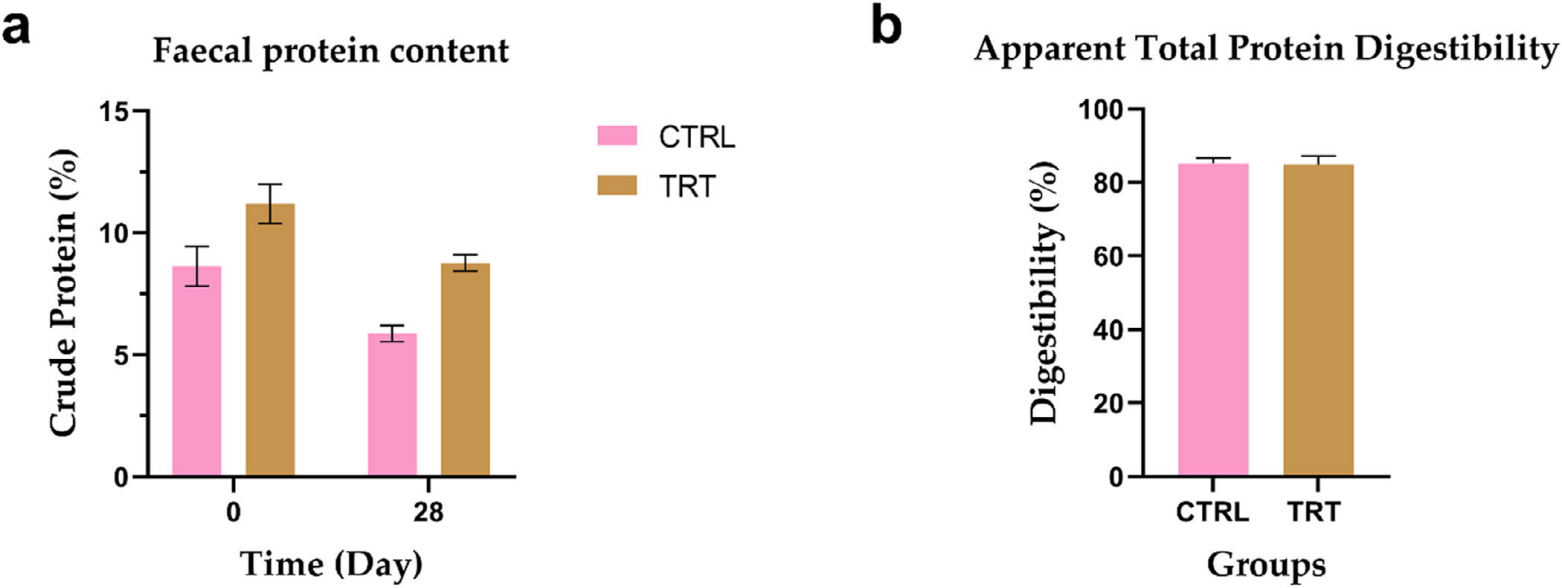
Faecal protein content and apparent total protein digestibility of control group (CTRL) and *P. vulgaris* *lec‐* *lpa* (TRT) at day 0 and day 28. Data are presented as means ± standard error (SE).

The faecal nitrogen content did not show statistically significant differences at day 0 and day 28. However, considering the entire experimental trial, the average faecal nitrogen content was significantly higher in the TRT compared to the CTRL (CTRL 1.60 ± 0.34%, TRT 1.16 ± 0.34%; *p* = 0.0014) (Figure 3). The apparent total tract protein digestibility revealed no differences between groups at the beginning (CTRL: 78.91 ± 17.71%, TRT: 91.69 ± 2.41%) and at the end of the trial (CTRL: 85.17 ± 3.91%, TRT: 84.89 ± 6.21%).

### 3.4 Serum Metabolic Profile

On day 28 of the trial albumin, A/G, total protein values were significantly lower in the TRT group compared to CTRL (*p* < 0.05). Urea, magnesium, aspartate aminotransferase and alkaline phosphatase values were significantly increased in the control group compared to TRT (*p* < 0.05) (Table 5).

**TABLE 5:** Serum metabolic profile of control (CTRL) and *P. vulgaris* *lec‐* *lpa* group (TRT) at 28 days of trial.

Serum metabolite	CTRL	TRT	SE	*p*‐value
Alanine aminotransferase (ALT/GPT), IU/L	51.00	49.29	3.68	0.6611
Albumin, g/L	36.91^a^	28.94^b^	1.37	0.0022
Globulin, g/L	15.94	19.33	2.12	0.3620
Albumin/Globulin (A/G)	2.33^a^	1.67^b^	0.16	0.0208
Total protein, mmol/L	52.86^a^	48.26^b^	1.08	0.0163
Total bilirubin, µmol/L	1.30	1.31	0.08	0.9909
Urea, mmol/L	2.24^a^	0.53^b^	0.29	0.0016
Glucose, mmol/L	6.73	6.59	0.22	0.5719
Total cholesterol, mmol/L	2.95	2.72	0.10	0.0856
Calcium, mmol/L	2.96	2.86	0.05	0.2016
Phosphorus, mmol/L	3.31	3.14	0.08	0.0857
Magnesium, mmol/L	1.19^a^	1.05^b^	0.04	0.0101
Aspartate aminotransferase (AST/GOT), IU/L	45.71^a^	36.86^b^	1.91	0.0066
Alkaline phosphatase (ALP), IU/L	278.14^a^	249.29^b^	15.3	0.0152

*Note*: Data are presented as means ± standard error (SE).

^a,b^ Means with diverse lowercase letters are significantly different (*p* < 0.05).

### 3.5 Microbiota Composition and Bacterial Community Diversity

**FIGURE 4:**
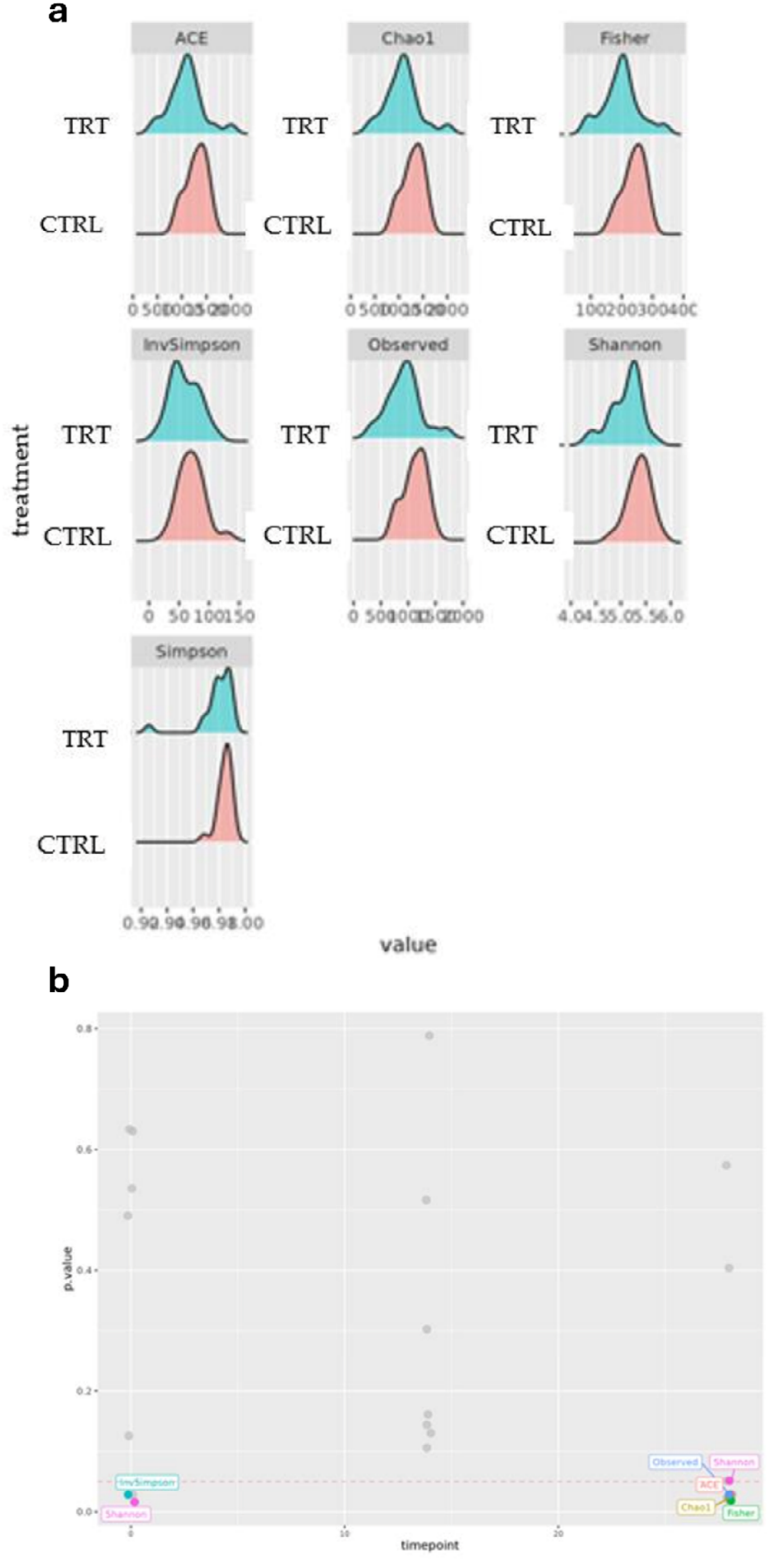
Alpha‐diversity indices relative to the comparison between the control (CTRL) and *P. vulgaris* *lec‐* *lpa* (TRT) groups during the experimental trial. (a) The indices ACE, Chao1, Fisher, InvSimpson, Observed, Shannon, Simpson presented for theCTRL and TRT groups and (b) figure presenting the significance level of the alpha diversity indices differences in TRT and CTRL group. The red line indicates the cut‐off value of 0.05.

The alpha diversity analysis showed significant values of Shannon, observed species, ACE, Fisher and Chao1 indices in the microbial composition of animals fed with *P. vulgaris* *lec‐* *lpa* at day 28 (*p* < 0.05) (Figure 4). In particular, alpha diversity indices were significantly lower in TRT at the end of the experimental trial (*p* < 0.05).

**FIGURE 5:**
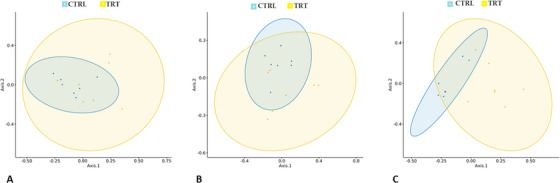
PCoA, Bray‐Curtis distance plot of the gut microbiota of weaned piglets fed with *P. vulgaris* *lec‐* *lpa* (TRT) and the control group (CTRL) at day 0 (A), day 14 (B) and day 28 (C).

The beta diversity was calculated with PERMANOVA analysis of the Bray–Curtis distances, which showed significant differences between experimental groups at day 14 (*p* = 0.016) and day 28 (*p* = 0.0039) (Figure 5).

**FIGURE 6:**
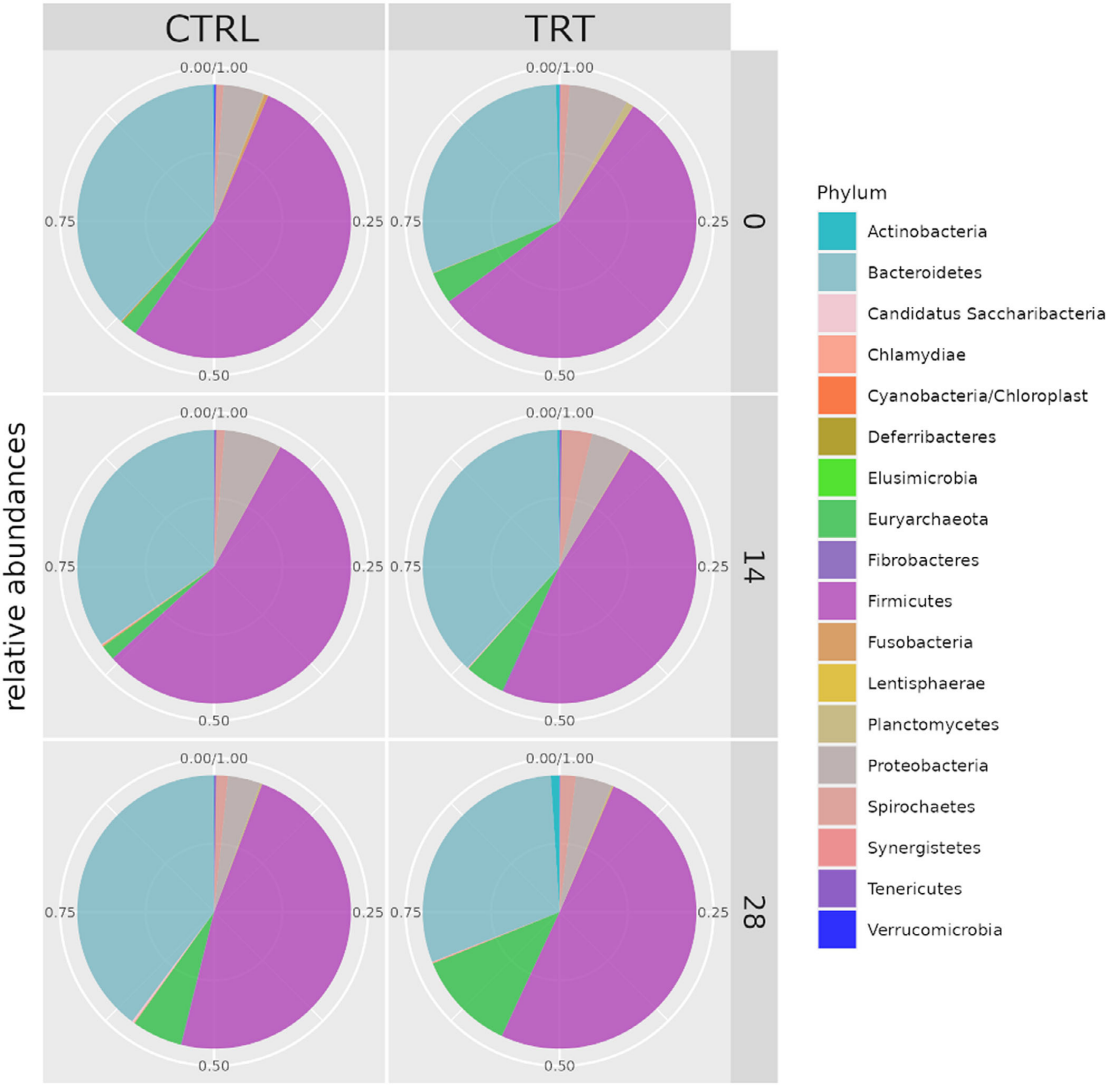
Relative abundances of phyla in comparison between control group (CTRL) and *P. vulgaris* *lec‐* *lpa* (TRT) at day 0, day 14 and day 28.

At the phylum level the most representative phyla were *Firmicutes*, *Bacteroidetes*, *Euryarchaeota* and *Proteobacteria* in both groups. At day 14 *Euryarchaeota* showed an increase in the TRT group, reducing the *Bacteroidetes* abundance, while in the CTRL group a decrease in *Firmicutes* and an increase in *Bacteroidetes* were registered. At day 28, *Euryarchaeota* increased and Bacteroidetes decreased in the TRT group; in the CTRL group Bacteroidetes decreased and *Euryarcheota* increased. (Figure 6).

At the family level, *Methanobacteriaceae* and *Spirochaetaceae* increased in the TRT samples at day 14 (*p* < 0.05); *Methanobacteriaceae* and *Clostridiaceae 1* increased significantly on day 28 in the TRT group (*p* < 0.05). At the genus level, *Mucispirillum, Holdemanella, Clostridium XIVb, Oribacterium, Chlamydia* were significantly higher at day 14 in CTRL group compared to TRT (*p* < 0.05). *Actinobacillus, Butyricimonas, Clostridium sensu stricto* and *Selenomonas* resulted higher in TRT at day 14 (p < 0.05). *Sutterella*, *Paraprevotella*, *Paraeggerthella*, *Oscillibacter*, *Mucispirillum* *Holdemanella*, *Fibrobacter*, *Clostridium XIVb*, *Clostridium XI*, *Butyricicoccus*, *Asteroleplasma*, *Anaerovibrio*, *Anaeroplasma* and *Alloprevotella* were more abundant in the CTRL group compared to the TRT group after 28 days (*p* < 0.05). On the opposite, *Turicibacter, Selenomonas, Roseburia, Romboutsia, Methanobrevibacter*, *Mitsuokella*, *Butyricimonas* and *Dialister* showed an increased abundance in TRT group at day 28 (p < 0.05).

## 4 Discussion

Soybean is the main source of plant‐based protein in animal feed. Although the European Union is self‐sufficient in many agricultural products, it has a deficit in soybean production, producing only 5% of the demand of the zootechnical sector (European Commission 2018). Therefore, the protein gap is filled by imports from Brazil, Argentina and the United States, in which GM crops, including soy, are highly advanced and productive (Nendel et al. 2023). Considering that, in the last decennium, many studies were focused on the evaluation of alternative protein sources to soybean that are safe for animals and environmentally sustainable (e.g., insects) (Ferri et al. 2023), algae (Frazzini et al. 2022) and encouraging local production (e.g., lupin) (Abraham et al. 2019; Struți et al. 2020), common bean (dos Santos et al. 2019), pea, chickpea and faba bean (David et al. 2024; Prandini et al. 2016). However, common beans raise concerns regarding the presence of antinutritional factors such as phytic acid, which limited their use due to the need to maximise the nutrients bio‐accessibility. In addition, the effect of increasing metal elimination through faeces has raised important sustainability concerns in pig farming (Provolo et al. 2018). Therefore, in this study, *Phaseolus vulgaris* *lec‐* *lpa* was used for the first time to evaluate the complete substitution of soybean meal, the principal protein source in animal diets, with 10% of *Phaseolus vulgaris* *lec‐ lpa* meal.

The obtained data on body weight showed a gradual and constant growth in both groups, but significantly higher weight in the CTRL was recorded at days 21 and 28 of the trial. As for pig farms, the control diet had soybean as the main protein source of plant origin; the weight gain of the control animals confirmed the high biological value of soya protein content. Results on ADG confirmed the same trend, revealing a faster growth in animals fed with a commercial diet with soybean. Considering the feed intake, no significant results were obtained from day 0 up to day 14 of the trial, suggesting that the substitution of 10% of *P. vulgaris* *lec‐ lpa* in the TRT did not affect the palatability. However, an increased feed intake was registered during the period from 15 to 21 days in CTRL compared to TRT as a result of a higher growth confirmed by an increased body weight during the same trial period in CTRL. The FCR values were lower in CTRL compared to TRT during the first week of the trial indicating a lower ability to exploit dietary nutrients in the TRT group. In fact, a limiting factor could be represented by the production of alpha‐amylase. In piglets, the concentration of alpha‐amylase increases with the age of the animal (Gao et al. 2022); thus, the post‐weaning phase represents an incomplete digestive maturity. In addition, the *P. vulgaris* *lec‐ lpa* genotype, selected for a reduced phytic acid and lectin content, may have retained a class of factors considered to be anti‐nutritional, including trypsin inhibitor (TI), which can inhibit protein degradation by interfering with trypsin activity, and alpha amylase inhibitor (aAI) (Barrett and Udani 2011). Thus, the nutrient exploitation may have been influenced by poor enzyme production and aAI activity that negatively interfered with starch digestion, reflecting in reduced feed conversion efficiency. Nevertheless, during the last week of the trial, an improved efficiency in the CTRL group compared to the TRT group was recorded. The obtained findings trends from the performance could be due to a different amino acid profile of the *P. vulgaris* *lec‐ lpa* bean compared to soybean. The amino acid composition of soybean protein has a high biological value, especially for its content of lysine, methionine, threonine, valine, isoleucine, leucine, tryptophan and phenylalanine (Kudełka et al. 2021). On the contrary, the amino acid profile of the common bean is considered suboptimal due to the low content of sulphur‐containing amino acids (Cominelli et al. 2022). Thus, the significative results of the CTRL group could probably be due to an improved efficiency of the diet. Although the performance in the soybean‐fed control was more promising, the partial replacement with *P. vulgaris* *lec‐ lpa* in the treatment still showed continuous growth of the animals and no clinical signs of intestinal disorders such as diarrhoea, suggesting an unaffected health status.

In order to assess the impact of diet on digestibility and nutrient absorption, faecal samples were evaluated for the protein digestibility and mineral content. *P. vulgaris* dietary inclusion did not influence apparent protein digestibility at 28 days of trial. Specifically, the nitrogen content, even if numerically higher in TRT, did not show significant differences considering the entire trial, suggesting a comparable utilisation of nitrogen provided by the diet. These data were supported by the absence of difference in faecal pH, which is considered a useful parameter to exclude protein malabsorption characterised by basic faeces due to the presence of ammonium as a fermentation end‐product. Moreover, pH analyses have excluded acid faeces, suggesting normal carbohydrate absorption (Maes et al. 2021).

Analysis of serum metabolic profile showed that among metabolite, albumin, albumin/globulin, total protein, urea, magnesium, aspartate aminotransferase (AST) and alkaline phosphatase (ALP) were significantly lower in the TRT group. Albumin, albumin/globulin and total protein are parameters of nutritional status (Bæk et al. 2020). More in detail, albumin is a protein produced by the liver; it represents 60% of plasma protein and is responsible for maintaining osmotic pressure in the blood. In this study, low albumin levels could be due to insufficient dietary protein intake or reduced hepatic synthesis of the protein. However, the administered diets were isonitrogenous, and the apparent protein digestibility was comparable for both diets, therefore, the obtained results on albumin, albumin/globulin and total protein could be related to a different protein quality.

Urea levels in the blood serum of the TRT were lower. Urea is a physiological waste product that the body produces during protein digestion. Therefore, blood urea can be considered an index of nitrogen utilisation efficiency (Yu et al. 2019). Lower urea levels could be associated with decreased protein catabolism due to reduced protein availability. AST and ALP are a class of hepatic enzymes produced by hepatocytes and localised in the cytoplasm. AST is a transaminase involved in the body's metabolic process; its high levels in the blood are usually related to liver dysfunction or oxidative stress. ALP catalyses the hydrolytic cleavage of phosphate esters of organic compounds in the presence of Mg as a cofactor. Considering that, lower magnesium levels could be associated with lower enzymatic activity of AST. These results, combined with lower ALP levels in the TRT group compared to the CTRL group, suggest a preserved liver function and a lower protein load.

Results on mineral content in faeces showed lower levels of Mg, Al, P, Ca, Cr, Mn, Fe, Co, Ni, Cu, Zn, Se, Cd and Pb in the TRT group, suggesting an increased gastrointestinal bioaccessibility of oligo and micronutrients. In particular, zinc and copper, previously used as growth promoters and antibiotic alternatives in feed for the control of enteric diseases, raised concerns about their environmental spread and the co‐selection of resistance genes in bacteria (Hejna et al. 2020; Rossi et al. 2014). Moreover, lower phosphorus levels in the TRT group suggested a positive impact of the inclusion of *P. vulgaris* *lec‐ lpa* on faeces pollutant content in manure and slurry. A lower environmental phosphorus release, along with decreased levels of zinc and copper, limits the eutrophication of soil and water (Colombo et al. 2024). In more detail, legumes are notably enriched in phytic acid. As an anti‐nutritional factor, the phytic acid precipitates during the digestive process in the form of phytate salts due to the intestinal pH (6–7) binding cations such as iron, zinc, calcium, and magnesium, limiting their availability (Bloot et al. 2023). Nevertheless, the *P. vulgaris lec‐lpa* genotype, poor in phytic acid, exhibited better mineral uptake performance than soybean, showing its potential to enhance mineral absorption and decrease faecal accumulation of contaminants. Considering the discussed results, a promising eco‐sustanable profile of *P. vulgaris lec‐lpa* emerges. Although it does reach the protein bioaccessibility of soybean, it shows interesting aspects. The production and use of *P. vulgaris lec‐lpa* could respond to the Green Deal policy on environmental preservation and sustainable agriculture with consequent implications on organic farming improvement and on a lower soybean import dependency (European Parliament 2020). Moreover, higher bioavailability for minerals, which are absorbed in only a small percentage of their inclusion (Madrid et al. 2013), enables greater efficiency and theoretically proposes an interesting strategy for reducing dependence on supplemented additives for feed formulation. Microbiota composition analysis allows us to underscore the complex interplay between bacteria and host that can be shaped with nutritional strategies (Han and Xiao 2020). Alpha and beta diversity suggested a different microbial population in the treated group compared to the control group.

In this study, the most represented bacterial phyla in both groups were *Firmicutes* and *Bacteroidetes*. The result is in line with previous studies confirming the presence of these phyla for 90% of the composition of the microbiome of piglets (Tang et al. 2020). Besides *Firmicutes* and *Bacteroidetes*, the phylum *Euryarcheota* increased in the TRT group compared to the CTRL over time. The increase in the phylum *Euryarcheota* could depend on the bean variety introduced in the diet. Indeed, *P. vulgaris lec‐lpa* belongs to the *Phaseolus* genus of the legume family and is known for high protein and unsaturated fatty acid contents, as well as high values of starch and non‐starch polysaccharides (Finetti et al. 2020). Among starches are classified the slowly digestible, the rapidly digestible and the non‐digestible. The last class is particularly interesting in modulating the gut microbiome; in fact, the non‐digestible starches act as a fermentative substrate (Dobranowski and Stintzi 2021). Considering this, even *P. vulgaris lec‐lpa* genotype is enriched with polysaccharides that are particularly complex for monogastric digestion but have promoted *Euryarcheota* growth. Methanogens belongs to *Euryarcheota* phylum (Wu et al. 2023). At the family level, *Metanobacteriaceae* were more represented in the treated group and increased during the 28 days of trial. In particular, a higher abundance of the genus *Metanobrevibacter* was identified in TRT piglets. Previous studies have confirmed the presence of *Metanobrevibacter* in pig faecal samples, suggesting that it is a commonly identifiable genus in pig faeces (Federici et al. 2015). The role of *Metanobrevibacter* in piglets has been recently investigated, unravelling that the metabolism of this bacterium supports the host by fermenting plant‐derived polysaccharides, producing short chain fatty acids (SCFAs), notably positive molecules for the maintenance of eubiosis. Moreover, the presence of *Metanobrevibacter* plays a beneficial role in microbial communication; archaea enhance the efficiency of hydrogen fermentation by consuming H_2_ produced by other bacteria. As a consequence, the hydrogen concentration in the gut is reduced while SCFAs increase, suggesting that these methanogens could also influence the bacterial colonisation environment (Yang et al. 2023). However, the most representative phylum of the TRT group was *Firmicutes*. The post‐weaning phase in piglets is considered a critical period as intestinal immune maturity develops and enteric diseases such as post‐weaning diarrhoea may occur (Dell'Anno et al. 2021). Considering microbiota analysis on day 14, *Selenomonas* and *Clostridium XIVb* and *Clostridium sensu stricto* increased in the treated group. The reported strains are opportunistic pathogens that can cause severe enteric disease (Nguyen et al. 2023). However, piglets did not exhibit episodes of diarrhoea during the trial, suggesting that the observed increase on day 14 did not impair the balance of the microbiota in terms of eubiosis. The *Holdemanella* genus, whose role has not yet been fully elucidated, appears to be a beneficial contributor to the health status because it is the major abundant genus found in healthy control microbiota samples (Hu et al. 2023; Liu et al. 2021; Zhang et al. 2022). On day 28, the mentioned strains remained abundant in the treated group; however, *Turicibacter*, *Roseburia*, *Dialister* increased significantly in TRT compared to CTRL. In this study, *Roseburia* increased in the TRT group at day 28; these results are confirmed by literature asserting that *Roseburia* increases in the intestinal microbiota of pigs after weaning (Choudhury et al. 2021). The abundance of *Roseburia* in weaned piglets’ microbiota is an index of improved digestive efficiency that becomes more similar to adult pig due to the better degradation of carbohydrates. In addition, *Roseburia* is a major producer of butyrate, an SCFA, relevant for limiting the opportunistic pathogens’ growth, improving gut barrier functions and counteracting inflammation (Tsiamis et al. 2017). The importance of an intestinal environment enriched in butyrate is also linked to the prevention of severe intestinal diseases such as chronic inflammation or colon‐rectal cancer (Singh et al. 2023). *Butyricicoccus* was significantly abundant in the CTRL group at day 28. This genus showed a protective and preventive action against pathogenic microorganisms, such as *C. perfringens*, and the occurrence of necrotic enteritis in poultry (Eeckhaut et al. 2016). Furthermore, in an in vivo study on a mouse model by Chang et al. (2020), strains of *Butyricicoccus*, such as *Butyricicoccus pullicaecorum*, were shown to improve the prognosis of colon‐rectal cancer mice by activating SCFA transporters and receptors. Among the phylum *Bacteroidetes*, the genera *Alloprevotella* and *Paraprevotella* were the most abundant at day 28 in the CTRL group, but they were not significantly increased on day 14. From literature, *Alloprevotella* and *Paraprevotella* are mainly present in the intestinal tract of weaned piglets (Chen et al. 2017). In particular, *Alloprevotella* is a succinate and acetate producing microorganism. Succinate and acetate act in synergy with butyrate in order to protect the integrity of the intestinal barrier and exert anti‐inflammatory activity (Y. Li et al. 2018). The abundance of the strains discussed demonstrated that *P. vulgaris lec‐lpa* could modulate the richness and diversity of the treated group's microbiota while maintaining a state of balance and eubiosis, confirmed by the good health status of the piglets and the increase in several bacteria that have been reported as beneficial for the host. Limitations of this study primarily concern the assessment of zootechnical performance parameters. While a minimum of three replicates per group is generally accepted for biological experiments, the evaluation of parameters such as ADFI and FCR ideally requires a higher number of pen replicates to ensure a more robust evaluation of these parameters. In our experimental design, the number of pens per group was limited to three due to the low number of available piglets, which may have reduced the statistical power for the zootechnical evaluation. However, all parameters measured at the individual animal level, including body weight, blood metabolites, faecal characteristics, and gut microbiota composition, were assessed on seven animals per group, thereby exceeding the conventional threshold considered for statistical analysis. This sample size was specifically selected to ensure sufficient power for the analysis of gut microbiota and faecal output, which were the primary objectives of the study rather than the evaluation of growth performance. Importantly, the innovation of this study lies in its investigation of gut microbiota modulation and faecal output in response to a low‐phytate, low‐lectin common bean dietary supplementation, as these aspects remain poorly explored in the literature compared to the more extensively studied growth performance effects with legume inclusion.

## 5 Conclusion

This study evaluated the effects of the substitution of soybean with 10% of *Phaseolus vulgaris* lec‐lpa in a post‐weaning piglet's diet. Although growth performances may be lower in TRT group piglets, *P. vulgaris lec‐lpa* is considered safe for piglets’ health and could represent an environmentally sustainable raw material. In fact, it exerted an improved mineral bioavailability, leading to a lowering mineral output in faeces and environmental dispersion. Consequently, *P. vulgaris lec‐lpa* could be an alternative protein source to imported GM soyabean improving local crop production, organic farming and European agriculture self‐sufficiency. In addition, *P. vulgaris lec‐lpa* exhibited an interesting microbiota modulatory activity in terms of richness and diversity. In particular, the increase in *Roseburia* and *Butyricimonas* is strictly related to SCFA production in the gastro‐intestinal tract contributing to the maintenance of intestinal integrity, promoting positive bacterial populations and limiting inflammatory phenomena.

## Author Contributions

**Luciana Rossi**: conceptualisation, methodology, investigation, writing – review and editing, supervision, funding acquisition, project administration. **Benedetta Canala**: methodology, investigation, Formal Analysis, Data Curation, Visualisation, Writing—Original Draft, Writing—Review and Editing. **Sara Frazzini**: methodology, investigation, formal analysis, data curation, writing – review and editing. **Matteo Dell'Anno**: methodology, investigation, formal analysis, data curation, visualisation, wring – review and editing. **Matteo Santoru**: methodology, investigation, formal analysis, data curation, wring – review and editing. **Irene Ferri**: methodology, investigation, formal analysis, wring – review and editing. **Francesca Sparvoli**: methodology, investigation, wring – review and editing. **Lucrezia Luciani**: methodology, formal analysis. **Bianca Castiglioni**: methodology, investigation, supervision. **Paola Cremonesi**: methodology, investigation, formal analysis, wring – review and editing. **Filippo Biscarini**: methodology, investigation, data curation, visualisation, Writing—Review and Editing. **Roberto Pilu**: methodology, investigation, supervision. **Martina Ghidoli**: methodology, fomal analysis.

## Ethics Statement

The authors confirm that the ethical policies of the journal, as noted on the journal's author guidelines page, have been adhered to and the appropriate ethical review committee approval has been received. The authors confirm that they have followed EU standards for the protection of animals used for scientific. The experimental trial was approved by the Animal Welfare Organization of the University of Milan (OPBA authorisation no. 4_2023).

## Peer Review

The peer review history for this article is available at https://www.webofscience.com/api/gateway/wos/peer‐review/10.1002/vms3.70597.

## Data Availability

The data presented in this study are available within the article and from the corresponding author upon reasonable request. The sequencing data generated in this study for microbiota analysis were deposited in the Zenodo repository and are publicly available at: https://zenodo.org/records/17046019.
